# Extrachromosomal DNA as a carrier of extra copies of oncogenes for heterogeneity and malignancy of glioma

**DOI:** 10.1016/j.apsb.2025.02.001

**Published:** 2025-02-03

**Authors:** Lin-jian Wang, Jianping Ye

**Affiliations:** a Institute of Trauma and Metabolism, Zhengzhou Central Hospital Affiliated to Zhengzhou University, Zhengzhou 450007, China; b Tianjian Laboratory of Advanced Biomedical Sciences, Academy of Medical Sciences, Zhengzhou University, Zhengzhou 450001, China

**Keywords:** Extrachromosomal DNA, Glioblastoma, Oncogene amplification, Heterogeneity, Prognosis

Extrachromosomal DNA (ecDNA), a circular DNA molecule first identified in 1965^1^, is now recognized as a key carrier of extra copies of oncogenes. Originating from chromosomal DNA, ecDNA primarily forms through mechanisms associated with chromosomal instability, such as DNA damage repair, chromothripsis, episome formation, and the breakage–fusion–bridge cycle. Ranging in size from 100 kb to several Mb, ecDNA is distinct from smaller extrachromosomal circular DNA (eccDNA) found in both tumor and normal eukaryotic cells. EcDNA confers cancer cells with significant advantages in proliferation and energy metabolism, while also contributing to tumor heterogeneity and malignancy. Despite growing recognition, the full characterization and functional roles of ecDNA remain incomplete, with critical questions regarding its biological significance, inheritance mechanisms, and therapeutic potential.

Recently, the eDyNAmiC project, led by Paul Mischel, published three groundbreaking original studies concurrently in *Nature*, revealing ecDNA's more profound impact on cancer than previously understood^2^, ^3^, ^4^. These studies highlight: (1) ecDNA's widespread distribution across various cancer types, with a higher prevalence than previously recognized^2^; (2) its contribution to cancer progression through multiple mechanisms and its “novel inheritance pattern that challenges traditional genetic principles”^3^; and (3) its identification as a promising therapeutic target^4^. Given its higher detection rates in glioblastoma (GBM)^2^^,^^5^, we focus on current understanding of ecDNA in GBM.

## 1 EcDNA in tumor malignancy

A pan-cancer analysis previously identified ecDNA in 14.3% of tumor samples^5^. The eDyNAmiC project further reported that 17.1% of tumor samples in the 100,000 Genomes Project (100kGP) harbor ecDNA, with the highest prevalence in liposarcoma (54.9%), GBM (49.1%), and HER2-positive breast cancer (46.4%) patients2. While ecDNA is implicated in early tumor transformation^6^, the eDyNAmiC project further found that its detection correlates with tumor stage and is more prevalent after targeted and cytotoxic therapies^2^. This suggests that ecDNA plays a critical role in early tumorigenesis and is associated with increased malignancy and therapeutic resistance^2^^,^^6^.

**Figure 1:**
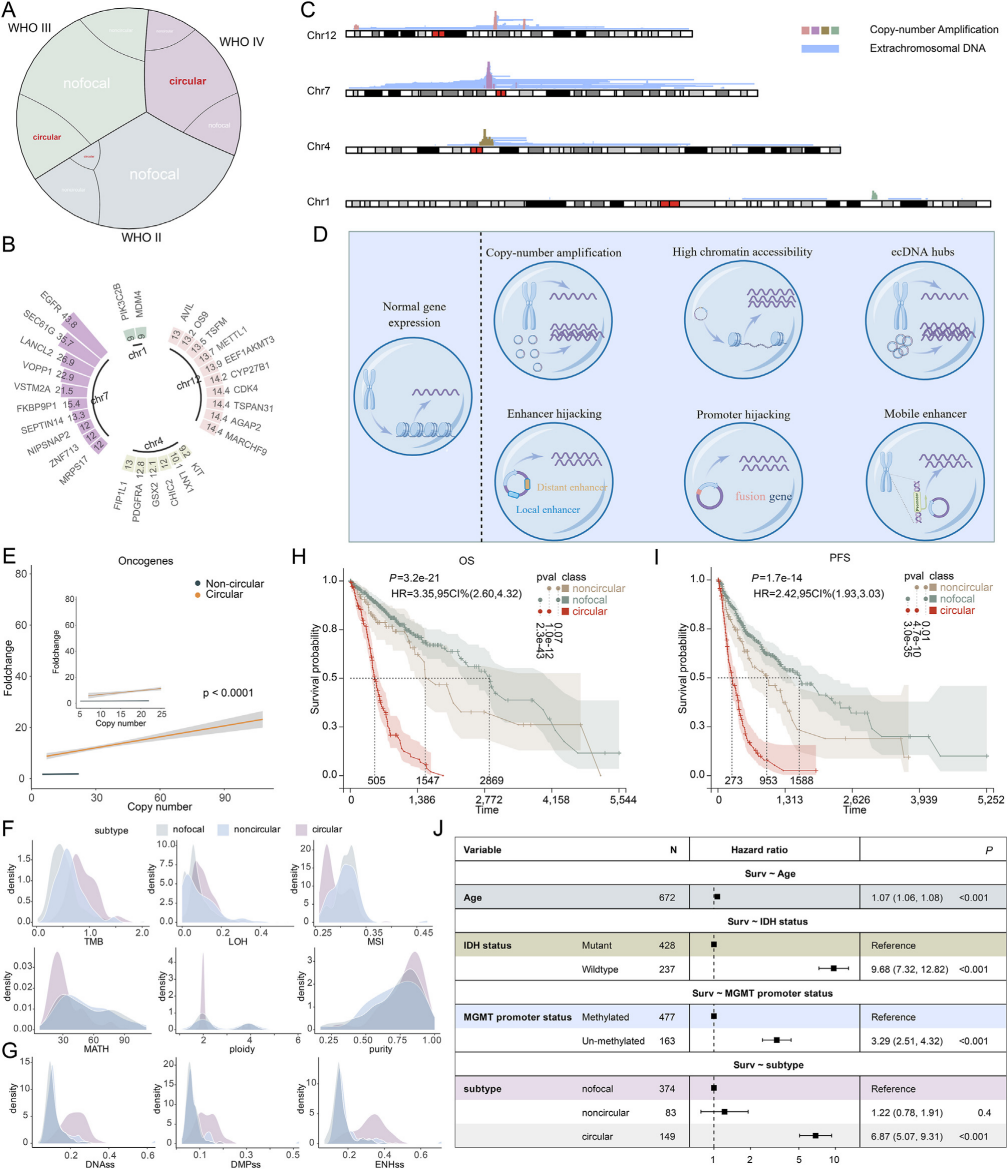
EcDNA contributes to heterogeneous and malignant progression of gliomas. (A) Analysis of gene amplification patterns in the TCGA GBMLGG cohort (*n* = 606). Expression and clinical data from the merged GBMLGG dataset in The Cancer Genome Atlas (TCGA) were downloaded from the UCSC Xena browser. Samples lacking survival or WHO grade information were excluded, yielding a dataset of 674 glioma patients. Subsequently, ecDNA data from a study by Hoon Kim^5^ were incorporated, resulting in a final cohort of 606 glioma patients included in this analysis. (B) Top amplified genes in GBM. (C) Karyotype plot showing the chromosomal distribution of amplified genes and ecDNAs-carried genes in GBM. (D) Overview of the mechanisms regulating gene expression in ecDNA. (E) Comparative analysis of expression levels between linear-amplified and circular-amplified oncogenes. (F) Assessment of tumor heterogeneity in the TCGA GBMLGG cohort. (G) Assessment of tumor stemness in the TCGA GBMLGG cohort. (H) Overall survival analysis comparing unamplified, linear-amplified, and circular-amplified groups (*n* = 606). (I) Progression-free survival analysis among unamplified, linear-amplified, and circular-amplified groups (*n* = 606). (J) Cox analysis demonstrating that ecDNA serves as an independent risk factor for glioma (*n* = 674).

To understand ecDNA activity in GBM, we analyzed gene amplification patterns within the GBMLGG cohort from The Cancer Genome Atlas (TCGA)^5^. Our analysis revealed a strong association between ecDNA amplification and glioma malignancy grades: WHO grade II: 85.6% unamplified, 12.6% non-circular amplification, 1.8% circular amplification; WHO grade III: 62.75% unamplified, 15% non-circular amplification, 22.3% circular amplification; WHO grade IV: 21.2% unamplified, 13.1% non-circular amplification, 65.7% circular amplification (Fig. 1A^5^). These findings suggest that ecDNA abundance increases with glioma grade, further implicating ecDNA in the progression of higher-grade malignancies.

## 2 Detection methods for ecDNA

EcDNA detection methods can be grouped into imaging-based and high-throughput sequencing (HTS)-based approaches^7^. Imaging techniques, such as electron microscopy, fluorescence in situ hybridization, and live-cell imaging with ecTag, are effective but are limited by the need for metaphase preparation, prior DNA sequencing, and low throughput. In contrast, HTS-based methods, including paired-end whole-genome sequencing and long-read sequencing, offer higher throughput and accessibility, making them more commonly relied upon in ecDNA research. However, the development of clinically viable and cost-effective ecDNA detection tools remains a challenge.

## 3 ecDNA as a vector for gene amplification

Previous studies have shown that ecDNA frequently amplifies driver oncogenes in tumors^8^. The 100kGP project revealed that while ecDNAs predominantly amplify oncogenes, they also carry immunomodulatory genes. Additionally, certain ecDNAs have been identified as carriers of only regulatory elements, such as promoters, enhancers, and lncRNAs^2^, expanding our understanding of ecDNA's composition and functional role in the regulation of gene expression. In the TCGA GBMLGG cohort, ecDNA frequently carries genes located on chromosomes 1, 4, 7, and 12, including well-known oncogenes such as CDK4, MDM2, SOX2, and EGFR, all recognized as tumor drivers by the GISTIC algorithm and/or listed in OncoKB™ (Fig. 1B and C). EcDNA also carries immunomodulatory genes like KDR and LAG3, which contribute to immune evasion.

## 4 ecDNA as a regulator of gene expression

Oncogenes amplified on ecDNA exhibit markedly higher transcript levels, compared to the same genes when not amplified by circularization. EcDNA amplifies oncogene expression through both gene copy number amplification and altered regulatory mechanisms (Fig. 1D)^8^. Lacking centromeres and positional constraints, ecDNA undergoes asymmetric segregation during cell division, leading to rapid increases in copy number. Even after normalizing copy number, ecDNA consistently drives higher oncogene expression due to reduced heterochromatin compaction, differential replication timings, and unique histone modifications^8^. For instance, MYC encoded on ecDNA is highly accessible both in the G1 phase and even during metaphase, placing it among the top 1% of genes expressed in cancer genomes^8^.

The circular structure of ecDNA facilitates the spatial proximity of DNA elements and oncogenes, enabling a phenomenon known as enhancer hijacking. In neuroblastomas, MYCN overexpression is driven not only by local co-amplified enhancers but also by the hijacking of distal enhancers, compensating for the loss of local regulatory elements^9^.

Post-mitosis, ecDNAs with different cargoes can interact in trans to form ecDNA hubs, which colocalize with RNA polymerase II, serving as active transcription sites. Regulatory ecDNAs identified by the eDyNAmiC project further suggest cross-regulatory interactions within ecDNA hubs^2^. Interestingly, these hubs allow ecDNAs to cooperatively inherit traits^3^, maintaining effective ecDNA interactions amidst random segregation, thereby amplifying oncogene expression. As a result, ecDNA transcription is often so rampant that it can cause significant transcription–replication conflicts in cancer cells^4^.

In the TCGA GBMLGG cohort, oncogenes located on ecDNA showed significantly higher expression than those on chromosomal loci, even after copy number normalization (Fig. 1E), highlighting the critical role of ecDNA in driving oncogene overexpression and cancer progression.

## 5 ecDNA as an evolutionary driver of tumor heterogeneity

EcDNA amplification is dynamic, evolving particularly in response to therapy-induced selection pressures^10^. Its prevalence increases after targeted therapy and cytotoxic treatments^2^, with new ecDNA species emerging during tumor recurrence. The random inheritance of ecDNA contributes to intratumoral heterogeneity, allowing tumors to adapt by selecting advantageous clones^11^. Notably, the eDyNAmiC project discovered that ecDNA species can actually be coordinately inherited, facilitating the maintenance of cooperative interactions that enhance tumor adaptation and evolution^3^. Additionally, ecDNA is prone to hypermutation, further amplifying intratumoral heterogeneity^12^.

In the TCGA GBMLGG cohort, ecDNA-positive samples exhibited higher tumor mutational burden and loss of heterozygosity compared to gene-unamplified and linear-amplified samples (Fig. 1F). Microsatellite instability (MSI) was significantly lower, consistent with the well-established pan-cancer pattern of mutual exclusivity between ecDNA amplification and MSI. Interestingly, we observed that ploidy was not elevated in ecDNA-positive samples, and that these samples exhibited increased purity, a result that deviates from typical pan-cancer trends (Fig. 1F). Moreover, ecDNA-positive samples also displayed enhanced tumor stemness, further highlighting the complex role of ecDNA in driving tumor heterogeneity and evolution (Fig. 1G).

## 6 ecDNA as a prognostic risk factor

EcDNA is strongly linked to poor prognosis due to its roles in oncogene amplification, tumor evolution, immune evasion, and therapy resistance^2^^,^^5^. In the TCGA GBMLGG cohort, ecDNA-positive samples exhibited significantly shorter overall survival and progression-free survival, compared to gene-unamplified and linear-amplified samples (Fig. 1H and I). No significant differences were observed between unamplified and linear-amplified groups, suggesting that ecDNA-driven amplification has a more profound impact on glioma progression than linear amplification alone. Cox regression analysis further confirmed the clinical relevance of ecDNA, identifying it as an independent risk factor for glioma prognosis (Fig. 1J), whereas linear amplification did not emerge as an independent prognostic factor.

## 7 Perspectives

EcDNA has been implicated in mechanism of drug resistance through rapid genomic rearrangements^2^. Its presence may also hinder immunotherapy effectiveness by amplifying immunosuppressive genes, emphasizing ecDNA's potential as a therapeutic target. However, targeting specific oncogenes located on ecDNAs, such as EGFR, may be inadequate, as their numbers can fluctuate or reintegrate into chromosomes under selective pressure, complicating treatment outcomes^10^^,^^13^. Disrupting ecDNA production and maintenance could offer a promising approach to overcoming ecDNA-driven resistance to targeted therapies.

One potential strategy is to target the formation of ecDNA via chromothripsis, followed by non-homologous end joining (NHEJ) and PARP-dependent repair pathways. Chemotherapeutic agents like methotrexate and vemurafenib have shown potential to induce chromothriptic rearrangements. Combining these treatments with DNA repair inhibitors may help prevent tumors from becoming more aggressive or resistant^10^. Additionally, recent research from the eDyNAmiC project indicates that ecDNA transcription increases transcription–replication conflicts, pushing cells into S-phase checkpoints when DNA replication remains incomplete. Targeting this conflict could exploit synthetic lethality as a strategy to combat cancer^4^.

Further exploration of ecDNA vulnerabilities is necessary to develop effective therapies. However, attempts to target ecDNA carry the risk of promoting the emergence of ecDNA-negative clones, potentially leading to treatment resistance, reminiscent of the challenges faced with kinase inhibitors. These considerations underscore the need for comprehensive preclinical trials to evaluate the effectiveness of ecDNA-targeted therapies, especially in combination with other treatment modalities.

## Author contributions

Lin-jian Wang drafted the manuscript. Jianping Ye conceptualized the study and revised the manuscript.

## Conflicts of interest

All authors state that they have no conflicts of interest.
